# Description of the Use of Plasma Exchange in Dogs With Cutaneous and Renal Glomerular Vasculopathy

**DOI:** 10.3389/fvets.2018.00161

**Published:** 2018-07-19

**Authors:** Ragnhild Skulberg, Stefano Cortellini, Daniel L. Chan, Giacomo Stanzani, Rosanne E. Jepson

**Affiliations:** ^1^Department of Clinical Science and Services, Royal Veterinary College, Hertfordshire, United Kingdom; ^2^Division of Medicine, Bloomsbury Institute for Intensive Care Medicine, University College London, London, United Kingdom

**Keywords:** plasmapheresis, plasma exchange, thrombotic microangiopathy, dialysis, apharesis

## Abstract

Cutaneous and renal glomerular vasculopathy (CRGV) is a rare disease affecting dogs, with a recent apparent increase in prevalence since 2012 in the UK. This disease is characterized by a vasculopathy affecting small vessels of the kidney and skin, leading to thrombotic microangiopathy. The underlying etiology remains unknown although clinicopathological and histological findings resemble features of certain forms of thrombotic microangiopathy in people, for which plasma exchange (PEX) is considered an important component of therapy. The objective of the present study is to describe the use of PEX as adjunctive treatment in dogs diagnosed with CRGV. A retrospective review of dogs diagnosed with CRGV between 2014 and 2016 treated with PEX was performed. Clinical records were reviewed and data relating to signalment, diagnostic tests and management strategies were summarized. Information and complications relating to PEX were recorded. Six dogs were diagnosed with CRGV (*n* = 2 ante-mortem, *n* = 4 post-mortem) and underwent PEX as part of their therapy. All dogs had cutaneous lesions and were azotemic with oliguria or anuria. All dogs underwent at least one PEX cycle; one dog had a single cycle PEX, three dogs two cycles PEX, and two dogs had one cycle PEX and one cycle of prolonged intermittent renal replacement treatment. Complications seen during PEX therapy included hypothermia (*n* = 4), tachycardia (*n* = 2), hypotension (*n* = 2), and hypocalcemia (*n* = 6). Two dogs survived to discharge, the remaining four dogs were euthanized. The positive outcome in two dogs treated with PEX despite the reported high mortality rate once acute kidney injury with oliguria/anuria occurs does not confirm success of this treatment. However, survival in two dogs that were initially oligoanuric highlights that further consideration and evaluation of PEX for this patient group is warranted for this specific disease. Additional studies are urgently needed to identify the underlying etiology of CRGV before more targeted therapies can be developed. Based on our findings, further evaluation of the role of PEX in this specific disease are warranted.

## Introduction

Thrombotic microangiopathy (TMA) syndromes have been described in people and in dogs with the hallmark of these conditions being endothelial damage and subsequent vascular occlusion leading to anemia, thrombocytopenia and organ dysfunction (1). Thrombotic microangiopathy has been described in isolated cases and was the presumed cause of an outbreak affecting over 100 greyhounds in the USA (2) following histopathological examination of cutaneous and renal tissue in 10 of these cases. One of the most common conditions associated with TMA in dogs has been described as cutaneous and renal glomerular vasculopathy (CRGV) (2–5). This is a serious and life-threatening condition with no underlying cause yet identified with limited therapeutic options available.

A recent retrospective study in dogs in the UK described both the clinical and histopathological findings associated with this condition in detail; patients typically presented with ulcerative skin lesions affecting the distal limbs, variable degrees of anemia, thrombocytopenia, and developed acute kidney injury (AKI) and kidney failure within 7–10 days (5).

In people, TMA can be caused by two main diseases: Thrombotic Thrombocytopenic Purpura (TTP) and Hemolytic Uremic Syndrome (HUS). Both TTP and HUS can manifest in acquired and congenital forms (1). Hemolytic Uremic Syndrome can be categorized as infection-associated HUS (*E. coli* Shiga toxin), atypical HUS (aHUS; dysregulation of alternative complement pathway), secondary HUS (e.g., collagen vascular disease, drug-associated, autoimmune diseases, disseminated malignancy and malignant hypertension) and idiopathic HUS (1, 6, 7) although contributing factors in affected individuals across these forms may overlap. The features of TMA can also be identified in patients with disseminated vascular coagulation (7). Thrombotic thrombocytopenic purpura has been related to the metalloprotease enzyme, ADAMTS13 deficiency. This protease is involved in the cleavage of ultra-large von Willebrand factor into smaller subunits. The most common cause of reduced levels of ADAMTS13 is its binding to IgG autoantibody present in TTP (8).

In the pathogenesis of TMA syndromes, the presence of specific circulating blood constituents is common, offering potential targets for therapy. Therapeutic plasma exchange (PEX), also known as plasmapheresis or total plasma exchange, consists of the removal of the patient's plasma (and its constituent components) via centrifugation or filtration through an extracorporeal circuit and its replacement with allogenic plasma transfusion, human serum albumin, isotonic crystalloids or synthetic colloids (9).

Therefore, the goal of PEX in this context is to remove molecules responsible for the pathogenesis of the microangiopathy, such as autoantibody-inhibited ADAMTS13 in TTP, or complement regulators [e.g., factor H (CFH) and membrane cofactor protein (MCP)], in aHUS (8). Plasma exchange has been shown to increase survival from 10 to 78% in people with TTP (1). Based on the American Society for apheresis' guidelines, PEX therapy is considered a first-line therapy (Category I) in people with clinicopathological signs, complement-mediated TMA (Factor H autoantibodies), drug-associated TMA (e.g., ticlopidine) and TTP (7, 8, 10, 11).

People with HUS often have a more severe degree of kidney injury compared to TTP, therefore PEX is recommended alongside early treatment with renal replacement therapy in the attempt to halt progression to irreversible kidney injury (8).

The clinical use of PEX in dogs has been previously described in several conditions such as systemic lupus erythematosus (12), hyperviscosity syndrome (13, 14), myasthenia gravis (15), immune-mediated hemolytic anemia (16), treatment of meloxicam and ibuprofen overdose (17, 18), and kernicterus (19). The management of patients with clinically suspected CRGV in the UK has been challenging given that there is no known underlying etiology and there is severe morbidity associated with the condition. In a recent report of dogs with suspected CRGV (5), all dogs that developed azotemia and oliguria/anuria died or were euthanized regardless of the treatment they received. Currently, a definitive diagnosis of canine CRGV is only made at post-mortem examination by renal histopathology and no therapy has been shown to prevent progression of the disease or improve outcome (5). In the absence of renal histopathological confirmation, compatible clinical presentation, clinicopathological changes and dermal histopathology are strongly supportive of a diagnosis of CRGV. Based on the potential similarity in pathophysiology between canine CRGV and conditions in people such as TTP or aHUS, PEX was hypothesized as a novel therapeutic option for affected dogs (5). The aim of this study was to retrospectively describe the use of PEX, report associated complications and outcome in six dogs clinically diagnosed with CRGV.

## Materials and methods

Dogs presented to the Queen Mother Hospital for Small Animals (QMHA) between January 2014 and December 2016 and diagnosed with CRGV were included in the study. Eligible dogs were identified by searching the hospital medical database using the terms “CRGV” and “plasmapheresis.” Inclusion criteria were dogs clinically diagnosed with CRGV receiving at least one cycle of PEX. A diagnosis of CRGV was made on the basis of renal histopathology if post-mortem examination had been performed or on the basis of compatible historical data, clinical signs including ulcerative skin lesions, hematological and biochemical findings and dermal histopathology compatible with the description provided by Holm et al. 2015 (5). Cases with an uncertain diagnosis of CRGV were excluded.

Medical records were reviewed in order to collect the following data for each patient: signalment, clinical signs, physical examination abnormalities, hematology using ADVIA 212Oi® (Siemens Healthcare, UK), serum biochemistry using iLab 600® (Instrumentation Laboratory, UK), activated partial thromboplastin time, prothrombin time using coagulation profile, ST Hemostasis Analyzer (Diagnostica Stago Ltd., UK) or IDEXX Coag Dx™ (IDEXX Laboratories, UK), urinalysis (including dipstick, urine protein creatinine ratio (UPC), sediment analysis and culture), imaging (abdominal ultrasonography, thoracic radiography), infectious disease including detection for Leptospirosis antibodies by using microscopic agglutination test, (Leptospirosis MAT®; IDEXX Laboratories, UK), Leptospirosis polymerase chain reaction test to detect Leptospira spp. (Leptospira spp. RealPCR Test® IDEXX Laboratories, UK) and test for tick-borne diseases by using SNAP® 4Dx® (IDEXX Laboratories, UK). Histopathological data from biopsies of skin lesions or post-mortem examination were reviewed. Clinical records were evaluated for information relating to the PEX therapy including the specific dialysis catheter used, type of anticoagulant provided, replacement fluids, each patient's PEX prescription, number of cycles, duration of cycles, requirements of additional transfusion of blood products, complications and outcome.

Descriptive statistics are provided and where indicated, results are reported as median (range).

## Results

Six dogs met the inclusion criteria during the study period. There was one dog diagnosed with CRGV during the study period which did not undergo PEX and was thus excluded. Two dogs were female entire and 4 dogs were female neutered. The median age was 4 years (10 months to 8 years) and median body weight was 21.8 kg (range 11.2–27). Breeds represented included 2 Labradors Retrievers, 2 mixed breed dogs, 1 Cocker Spaniel and 1 Dalmatian.

### Clinical signs

All dogs were reported to have developed cutaneous lesions. The length between observation of the cutaneous lesions and admission to the primary veterinary surgeon was 6 days (range 1–9); 5/6 dogs were reported to have progressive lameness and a stiff gait. Other presenting complaints included lethargy (*n* = 3, case 2, 4, 6), inappetence (*n* = 3, case 2, 3, 4), hemorrhagic diarrhea (*n* = 2, case 4, 6) and vomiting (*n* = 1, case 6). All dogs had been hospitalized for a median of 3 days (range 1–7) at the referring practice prior to admission to our center.

Clinical signs were reported to have acute onset prior to presentation to their primary veterinary surgeon and rapid clinical deterioration was reported in all dogs.

At presentation, the abnormalities on physical examination and initial assessment included cutaneous lesions, which were mainly identified at the extremities [*n* = 6, respectively pelvic limbs (*n* = 4, case 1, 2, 3, 4) and thoracic limbs (*n* = 2, case 5, 6)], ventrum (*n* = 1, case 3), and on the head with both cutaneous and oral lesions (*n* = 1, case 1). All dogs were normothermic at time of admission [median 38.5°C, (range 37.7–38.9°C)].

Three dogs were tachycardic [case 1, 4, 5; respectively 148 (case 1) and 120 (case 4 and 5) beats per minute] at time of presentation. These dogs were normotensive with evidence of volume overload. In total 5/6 dogs had evidence of volume overload. Volume overload was identified as weight gain in comparison to the weight reported in the history when healthy (*n* = 5), the presence of cutaneous edema (*n* = 3) or cavitary effusion identified with ultrasonography (*n* = 4, 3 dogs had peritoneal effusion and 1 dog had both pleural and peritoneal effusion). All six dogs were either oliguric (*n* = 3, cases 4, 5, 6) or anuric (*n* = 3, cases 1, 2, 3) at the time of referral. Four dogs were hypertensive using criteria established by Brown et al. (20) at time of presentation, diagnosed using the Doppler technique (cases 2, 3, 4, 5; respectively 225, 252, 180 and 170 mmHg).

### Clinicopathological findings

Clinicopathological findings for all dogs are summarized in Tables 1, 2. Serum biochemistry demonstrated that all dogs were azotemic [creatinine 301 μmol/L, 144–676 μmol/L (reference interval (RI) 59–138 μmol/L); urea 35.9 mmol/L, 13.2–72.6 mmol/L (RI 3–9.1 mmol/L)] and hyperbilirubinemic [4.85 μmol/L, 3.4–96.7 μmol/L (RI 0–2.4 μmol/L)] and that 5 dogs were hypoalbuminemic [22.1 g/L; 19.7–23.4 g/L (RI 28–39 g/L); Table 1). Dogs were graded according to the International Renal Interest Society (IRIS) AKI grading system (21): Grade III (*n* = 4), Grade IV (*n* = 1), Grade V (*n* = 1) during the first 24–48 h of hospitalization. During the time of hospitalization, the median peak concentration of creatinine was 693.5 μmol/L (range 509–1199), which corresponded to progression to IRIS AKI grade IV (*n* = 5) and V (*n* = 1). The lowest concentration of albumin measured during hospitalization was 19.85 g/L (range 10–22.7).

**Table 1:** Admission serum biochemical and coagulation times for six dogs with cutaneous and renal glomerular vasculopathy at the tertiary referral center.

	**Reference interval**	**MEDIAN**	**IQR**	**Q1**	**Q3**	**Case 1**	**Case 2**	**Case 3**	**Case 4**	**Case 5**	**Case 6**
Total protein	49–71 g/L	**44.6**	**17**	38.78	51.05	**34.2**	**44.6**	**44.2**	**47.9**	**40.3**	60.5
Albumin	28–39 g/L	**21.40**	**7**	20.23	25.15	**19.7**	**20.4**	**22.7**	**23.4**	**21.4**	30.4
Globulin	21–41 g/L	**24.2**	**11**	17.8	25.9	**14.5**	24.2	21.5	24.5	**18.9**	30.1
Sodium	142–153 mmol/L	**147.0**	**3**	146.75	152.25	148	146	156	147	151	147
Potassium	3.9–5.5 mmol/L	**4.5**	**1**	4.08	5.18	4.1	4.6	5.1	4	5.4	4.5
Chloride	105–118 mmol/L	**112.0**	**11**	108.25	115.75	121	112	114	110	113	**103**
Calcium	2.13–2.7 mmol/L	**2.35**	**1**	2.01	2.4	2.36	2.13	2.14	2.35	2.53	**1.66**
Inorganic Phosphorus	0.8–2 mmol/L	**3.13**	**2**	2.39	4.1	**3.13**	1.92	**3.31**	**5.07**	**3.77**	**2.55**
Urea	3–9.1 mmol/L	**37.5**	**31**	21.08	46.43	**37.7**	**13.2**	**23.7**	**72.6**	**37.5**	**34.2**
Creatinine	59–138 μmol/L	**375**	**363**	185.25	484	**375**	**144**	**199**	**676**	**420**	**227**
Cholesterol	3.3–8.9 mmol/L	**4.5**	**1**	4.13	6.35	4.2	4.5	6.8	4.7	3.9	6.2
Total Bilirubin	0–2.4 μmol/L	**5.6**	**56**	3.4	41.58	**3.4**	**5.6**	**3.4**	**23.2**	**4.1**	**96.7**
Amylase	176–1,245 U/L	**1212**	**1290**	846	1,829.75	949	1188	537	**3416**	**1301**	1212
Lipase	72–1,115 U/L	**299**	**1728**	107.75	1,118.25	152	101	110	**3327**	299	382
ALT	13–88 U/L	**45**	**327**	40.75	275.75	41	40	**125**	45	**184**	**551**
ALP	19–285 U/L	**101**	**187**	80.5	214.25	43	101	110	93	174	**335**
Creatine kinase	61–394 U/L	**259**	**1100**	226.25	1070.25	259	179	260	1791	830	242
Prothrombin time	7.5–9.9 (11–17) s	**11.3**	**3**	9.8	13	8.5	12		14	11.3	11.1
Activated Thromboplastin time	11–21 (72–102) s	**16.3**	**108**	13.9	122	13.8	**109**		**135**	16.3	14

*The parameters outside the reference range have been highlighted in bold for six dogs with cutaneous and renal glomerular vasculopathy at the tertiary referral center*.

**Table 2:** Hematology analysis performed on admission in six dogs with cutaneous and renal glomerular vasculopathy at the tertiary referral center.

	**Reference Interval**	**Median**	**IQR**	**Q1**	**Q3**	**Case 1**	**Case 2**	**Case 3**	**Case 4**	**Case 5**	**Case 6**
RBC	5.5–8.5 1012/L	**4.25**	**3**	**3.9**	**6.57**	**3.95**	**3.9**	6.33	**3.9**	**4.55**	7.3
HCT	37–55%	**31.6**	**24**	**27.18**	**51.23**	**28.1**	**27.1**	50.9	**27.2**	**35.1**	52.2
PCV	37–55%	**29.5**	**26**	**26**	**51.75**	**26**	**26**	51	**26**	**33**	54
Hgb	12–18 g/dL	**10.7**	**8**	**9.25**	**16.88**	**9.1**	**9.3**	16.3	**9.7**	**11.7**	**18.6**
MCV	60–77 fL	**71.4**	**8**	**69.65**	**78**	71.3	69.5	80.4	69.7	**77.2**	71.5
MCH	19.5–24.5 pg	**25.15**	**2**	**23.7**	**25.73**	23.1	23.9	**25.8**	**24.8**	**25.7**	25.5
MCHC	32–36 g/dL	**33.8**	**3**	**32.33**	**35.63**	32.4	34.3	32.1	35.6	33.3	35.7
RDW	%	**12.8**	**2**	**11.93**	**13.78**	13.6	12.7	11.7	14.3	12.9	12
WBC	6–17 109/L	**13.12**	**6**	**12.02**	**17.68**	13	14.29	12.8	13.23	**27.86**	9.69
Neutrophils	3–11.5 109/L	**10.2**	**7**	**7.62**	**14.97**	9.9	12	10.5	6.62	**23.86**	7.95
Neutrophils %		**82**	**15**	**69.5**	**84.25**	76	84	82	50	85	82
Lymphocytes	1–4.8 109/L	**1.69**	**2**	**1.03**	**2.84**	1.95	1.43	1.28	3.84	2.51	**0.29**
Lymphocytes %		**10**	**11**	**7.5**	**18.5**	15	10	10	29	9	3
Monocytes	0.15–1.5 109/L	**1.18**	**1**	**0.76**	**1.92**	0.78	0.71	0.9	**2.65**	**1.67**	1.45
Monocytes %		**6.5**	**11**	**5.75**	**16.25**	6	5	7	20	6	15
Eosinophils	0–1.3 109/L	**0.13**	**0.2**	**0**	**0.2**	0.39	0.14	0.13	0.13	0	0
Eosinophils %		**1**	**1.5**	**0**	**1.5**	3	1	1	1	0	0
Basophils	0			**0**	**0**	0	0	0	0	0	0
Basophils %				**0**	**0**	0	0	0	0	0	0
Platelets	150–900 109/L	**80**	**66.2**	**26.25**	**92.5**	**80**	**80**	**80**	**15**	>**130**	**30**

*RBC, Red Blood Cells; HCT, Hematocrit; PCV, Packed Cell Volume; Hgb, Hemoglobin; MCV, Mean Corpuscolar Volume; MCH, Mean Corpuscolar Hemoglobin; MCHC, Mean Corpuscolar Hemoglobin Concentration; RDW, Red blood cell Distribution Width; WBC, White Blood Cells. The parameters outside the reference range have been highlighted in bold*.

Hematology revealed a non-regenerative anemia in 4 dogs [median PCV 26%, range 26–33% (RI 37–55%)] and thrombocytopenia in all dogs [median estimated manual platelet count was 75 × 10^9^/L (range 15–80 × 10^9^/L) (RI 150–190 × 10^9^/L); see Table 2]. None of the patients had received any previous blood product transfusion. Standard coagulation profile including activated partial thromboplastin time and prothrombin time were within reference intervals in all the five dogs in which they were tested. One of the six dogs did not have a standard coagulation profile performed (see Table 1).

Urine analysis (*n* = 6) showed a median urine specific gravity of 1.015 [range 1.009–1.021 (RI 1.018–1.050)], with varying degrees of proteinuria (1+, case 5; 2+, case 4 and 6; 3+, case 1, 2, 3). Granular casts were documented in 3/6 dogs (cases 1, 5, 6). Four of six dogs (case 1, 3, 5, 6) had an evaluation of UPC ratios performed, with a median of 2.95 [range 0.49–11.16 (RI < 0.5)]; of these only one dog had an inactive urine sediment analysis with low cellularity. Urine culture was performed in 4/6 dogs. The collection of urine via cystocentesis for bacterial culture was deemed contraindicated in two dogs due to severe thrombocytopenia. A urinary tract infection was diagnosed in 1 dog (case 1); this was suspected to be hospital-acquired following urinary catheterization and the following organisms were cultured: *Escherichia coli, Enterococcus* spp., and *Staphylococcus aureus*. This dog, at time of presentation, had a negative urine culture prior to urinary catheterization. When the diagnosis of a urinary tract infection was made following repeat urine analysis the antibiotic therapy was selected based on culture and sensitivity test results.

Testing for *Anaplasma phagocytophilum, A. platys, Borrelia burgdorferi, Ehrlichia canis and E. ewingii* antibodies and *Dirofilaria immitis* antigens was negative in the two dogs (case 2 and 6) tested by using SNAP 4DX. Leptospirosis testing was performed in all six dogs via MAT. Two dogs were also tested using urine PCR (case 2 and 4). Four dogs (case 1, 2, 3, 5) had Leptospirosis titers between 1:100 and 1:200, which are not suggestive of active infection and in the remaining two dogs all titers were negative (case 4 and 6) (22); rising titer MAT testing was not performed in any dog.

Histopathological evaluation of skin biopsies in all dogs revealed necroulcerative dermatitis with vasculitis and thrombosis. The main findings included extensive dermal ulceration with necrotizing folliculitis and perifolliculitis, and adnexal necrosis with occasional vasculitis. The lesions were largely centered to the hair follicles and adnexa. Two dogs (case 1 and 2) had a skin culture taken which revealed a growth of *Escherichia coli, Staphylococcus pseudointermedius, Streptococcus canis*, and *Pseudomonas aeruginosa*.

### Diagnostic imaging

Assessment for the presence of peritoneal effusion was performed by point-of-care ultrasound and peritoneal effusion was present in 4/6 dogs (cases 2, 3, 4, 5) which included presence of fluids in 1–2 out of 4 quadrants of the abdomen. A complete abdominal ultrasound was performed in all dogs. In 4/6 dogs (case 1, 3, 4, 5) an enlarged and edematous pancreas with mixed echogenicity was detected. One of these dogs (case 5) also had gallbladder wall edema reported. One dog (case 6) had a thickened gastric wall with loss of layering, likely due to inflammation. One dog (case 6) had changes to the descending colon, consistent with ongoing diarrhea and possible colonic ulceration, with a maintained intestinal wall layering and normal thickness. One dog (case 5) had a 3.5 mm cortical nephrolith detected in the right kidney, otherwise no ultrasonographic changes were reported in the kidneys of other dogs.

Thoracic radiography was performed in all dogs following placement of the dialysis catheter, with no abnormalities reported on intrathoracic structures. There was only 1 dog (case 5) that had a scant pleural effusion present at time of arrival on point-of-care ultrasound of thorax. Three dogs had echocardiographic assessment by a board-certified cardiologist (cases 2, 4, 5): one dog (case 5) had signs of aortic insufficiency and one dog (case 2) had a mild depression of systolic function and dilatation of the center ventricle.

### Medical management

Medical management from time of admission to the hospital prior to PEX therapy varied. Only one of the six dogs (case 6) received intravenous fluid therapy (Aquapharm 11®), at time of admission due the presence of clinical dehydration. All dogs received antimicrobials in the event the pathogenesis was related to an infectious cause. The following antimicrobials were administered: potentiated amoxicillin (Augmentin®) (*n* = 5 case 1, 2, 3, 4, 6), doxycycline (Ronaxan®) (*n* = 2, case 1, 2), marbofloxacin (Marbocare®) (*n* = 1, case 3), generic ampicillin (Ampicillin®) (*n* = 1, case 5), generic cefuroxime (Cefuroxime®, case 5) and generic cephalexin (Rilexine™) (*n* = 1, case 1). All dogs also received antiemetic therapy: maropitant (Cerenia®) (*n* = 6), metoclopramide (Emeprid®) (*n* = 5, case 1, 3, 4, 5, 6) and generic ondansetron (Ondansetron®) (*n* = 5, case 1, 2, 4, 5, 6). All six dogs received a proton pump inhibitor (Omeprazole®). Further medical management consisted of amlodipine (Amodip®) (*n* = 5, case 1, 2, 3, 4, 5) and pentoxifylline (Trental®, case 1, 2, 3, 5) (*n* = 4). Pentoxifylline was administered as a potential adjunctive treatment for the presumed presence of vasculitis. One dog (case 4) required anti-seizure medication with diazepam (Diazemuls®) and levetiracetam (Keppra®). Analgesia was required in 5/6 dogs: methadone (Synthadon®) (4/5 case 1, 3, 4, 5, 6) and buprenorphine (Buprecare®) (1/5, case 2).

Within the first 24 h of monitoring urine output (UOP), four dogs (case 1, 2, 3, 5) received furosemide (Dimazon®) either as a bolus or constant rate infusion in the attempt to improve urine production. Urinary output was quantified using an indwelling urinary catheter and a closed collection system in 4/6 dogs (cases 1, 2, 3, 5) and estimated through monitoring of body weight and assessment of fluid balance by assessing presence of cutaneous edema and cavitary effusions in all dogs. Prior to initiation of diuretic therapy, UOP measured in 4 dogs was 0, 0.25, 0.3, and 0.5 mL/kg/h. After initiation of diuretics the UOP increased to 1.55, 2.81, 2.87, and 3.64 mL/kg/h. This was the UOP measured during hospitalization at the time of PEX therapy. The two dogs without initial urinary catheterization were oliguric with minimal urination prior to first PEX cycle, and both dogs had a urinary catheter placed at the time of PEX. One of these dogs (case 6) was clinically dehydrated and received fluid therapy which resulted in polyuria with a median UOP 7.4 mL/kg/h (range 5.5–17.9 mL/kg/h) on day 2 of hospitalization. Another dog (case 2) became polyuric over a short period of time after initiation of diuretics.

### Advanced medical management–plasma exchange

The decision to perform plasma exchange was made within 24–48 h of hospitalization due to lack of clinical improvement, worsening azotemia and persistent oliguria or anuria despite advanced medical management as described.

**Figure 1:**
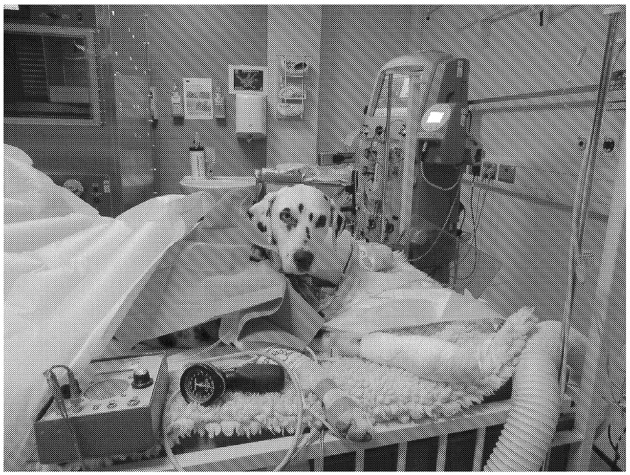
Patient undergoing a plasma exchange (PEX) cycle. Permission for publication of this image was obtained by the clients. In the background, the extracorporeal plasma filtration unit can be seen (Prismaflex®;Gambro).

**Figure 2:**
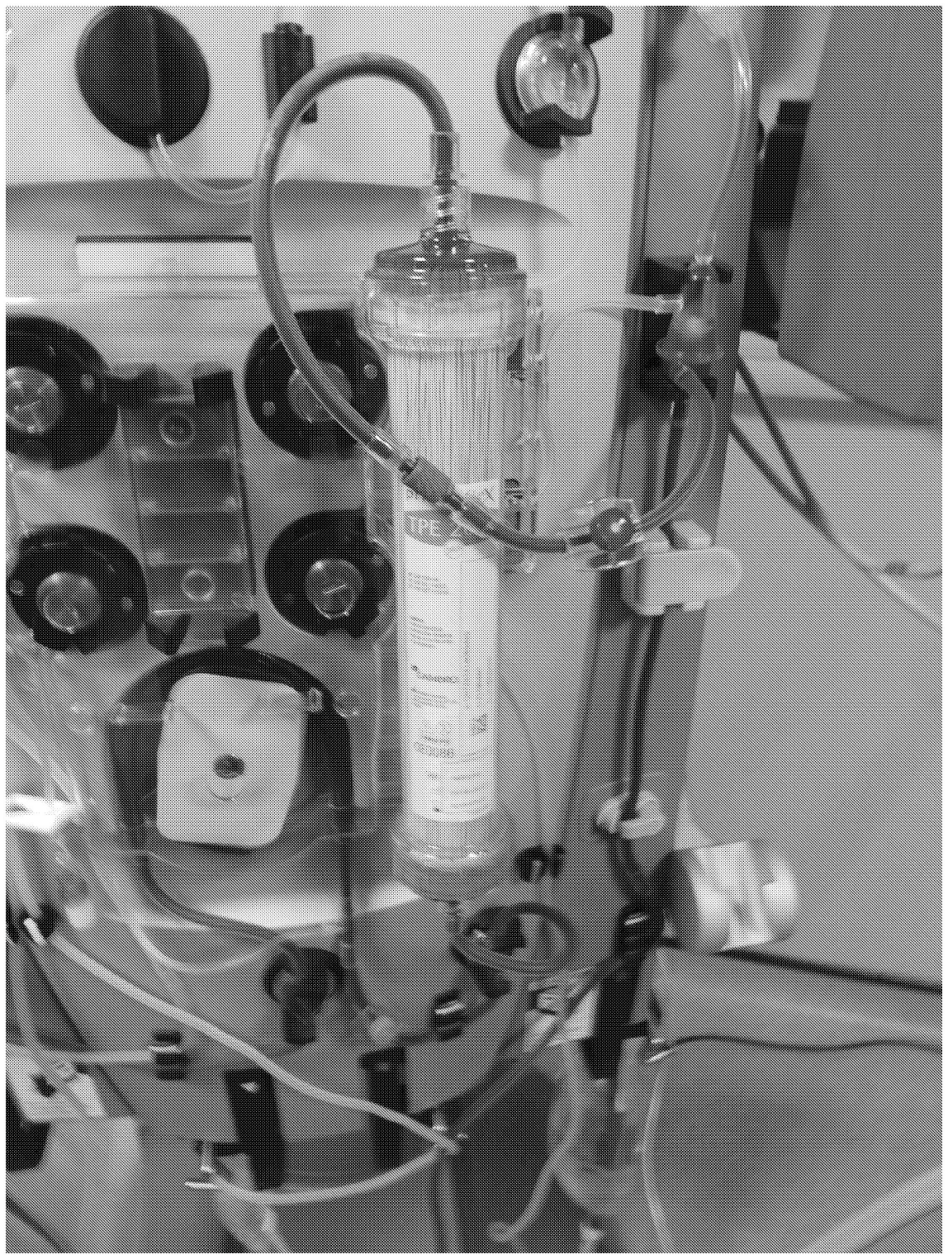
The extracorporeal plasma filtration system can be seen (Prismaflex® TPE 2000; Gambro).

Three dogs (case 1, 3, 4) underwent one PEX cycle and two of these dogs (case 3 and 4) also required a cycle of prolonged intermittent renal replacement treatment (PIRRT) with veno-venous hemodiafiltration to treat the severe azotemia and hyperkalemia the following day. The duration of PIRRT treatment was respectively 5 and 6.5 h, which resulted in a urea reduction ratio (URR) of 45.6 and 56%, respectively. The three other dogs (case 2, 5, 6) underwent two cycles of PEX within 48–72 h. Only case 1 received a single cycle of PEX, the remaining 4 dogs received two extracorporeal therapy cycles each (see Table 3). The PEX cycles were performed using an extracorporeal plasmafiltration system (Prismaflex® Software version 6.XX, Prismaflex® TPE 2000 with a 125 mL blood volume per set) and the PIRRT was performed using Prismaflex® Software version 6.XX, Prismaflex®; Prismasol®4; Gambro, UK) (see Figures 1, 2).

**Table 3:** Summary of the plasma exchange therapy in six dogs with cutaneous and renal glomerular vasculopathy at the tertiary referral center.

	**Median**	**IQR**	**Q1**	**Q3**	**Case 1**	**Case 2**		**Case 3**	**Case 4**	**Case 5**		**Case 6**	
Body Weight (Kg)	21.3	14	13.85	27.75	25.7	27.7		10.4	16.8	27.9		15	
Number of PEX cycles	1.5	1	1	2	1	2		1	1	2		2	
Duration of cycles (Hours)	3	1	2	3.25	3	3	2.5	3.5	4	3	2	2	2
PV Exchanged (ml)	1,440	957	898.5	1,667.5	1,710	1,688	1,647	495	1,249	1,631	1,426	817	980
Achieved PV exchange	1.9	1	1.45	2.38	2	1.8	1	3.3	2.5	1.6		1.8	1.4

*Kg, Kilogram; PV, Plasma Volume*.

Vascular access was achieved in all dogs by placement of a short-term, double lumen, dialysis catheter (Coviden™, Mahurkar™; Acute Dual Lumen Catheter Kit) in the jugular vein. The chosen size of the jugular catheter was based on the size of each dog, ranging from 11.5–13.5 Fr to 19–24 cm. Four dogs had the catheter placed in the right jugular vein and two dogs in the left jugular vein. The procedure was performed under general anesthesia using surgical aseptic conditions with modified Seldinger technique used. Anticoagulation for the PEX cycles was achieved with regional anticoagulation, infusing Citrate ACD-A (Haemonetics®) through the pre-blood pump in all 6 dogs.

Our citrate protocol consisted of administering Citrate ACD-A at a rate of 5.65 mmol citrate per liter of circulating blood for 30 min. Following this we aimed to reduce the citrate infusion to 1.69–2.26 mmol/L of blood. The citrate infusion was titrated to achieve an extracorporeal plasma ionized calcium concentration of < 0.35 mmol/L. Calcium gluconate (Calcium gluconate 10%) was administered diluted to 2.4% with NaCl 0.9% (Aquapharm 1®) at a starting rate (ml/h) of 0.4 times the citrate infusion (ml/h) and subsequently titrated to achieve a target plasma ionized calcium in the patient between 1.1 and 1.3 mmol/L (23).

The median Plasma Volume (PV) exchanged with PEX was 1.8 (range 1–3.3) with a median volume of 1426 mL (range 495–2,565 mL). The PV was calculated by the following equation; PV = (BW(kg) × 0.09) × (1-hematocrit) (16, 24). PEX was performed over a median time of 3 h (range 2.5–4) (see Table 3). Plasma removed was replaced with a combination of 0.9% saline (Aquapharm 1®) and stored (SFP) or fresh frozen plasma (FFP). For each cycle, the replacement fluid initially used was 0.9% sodium chloride before transitioning to FFP/SFP infusion. The median time before transitioning to plasma was 70 min (range 25–120). The plasma prescribed was blood-type matched for each recipient. Plasma administered accounted for 30.4% (range 16.3–48.8) of the total volume exchanged.

Complications observed during plasma exchange included hypocalcemia, hypothermia, sinus tachycardia and hypotension. All dogs developed hypocalcemia (median ionized calcium 0.68 mmol/L (range 0.55–0.86)). However, only one patient (case 6) developed clinical signs attributable to hypocalcemia, with facial twitching which resolved with an additional 1 mL/kg IV bolus of 10 % calcium gluconate. Hypothermia (<37°C) was identified in 4 dogs despite the use of active heating with forced air heating blankets from the outset (Bair Hugger model 505; Augustine Medical), and the use of a blood warmer on the extracorporeal circuit return line immediately after connection. Sinus tachycardia (heart rates of 189 and 190 beats per minute, respectively) was observed in 2 patients (case 3 and 6).

Two dogs (case 3 and 6) developed severe hypotension (Doppler systolic pressure 60 mmHg), one during a cycle of PEX and the other during a cycle of PIRRT. Case 6 developed hypotension during her first PEX cycle due to hypovolemia from bleeding from venipuncture and the catheter insertion sites. This patient was severely thrombocytopenic. The hypotension did not respond to fluid resuscitation [45 mL/kg bolus of Hartmann's Solution (Aquapharm 11®)] and required vasopressor therapy with a noradrenaline (Norepinephrine®) constant rate infusion. Later in the same PEX cycle, this patient developed neurological signs (obtundation and nystagmus) at the end of treatment cycle requiring interruption of PEX and the return of the extracorporeal blood to the patient. Due to ongoing bleeding this patient was further stabilized with a transfusion of packed red blood cells (pRBC).

The second dog (case 3) developed hypotension during her PIRRT cycle which did not respond to fluid resuscitation, also requiring noradrenaline constant rate infusion. This patient later developed neurological signs (mitotic pupils and loss of muscle tone) which was stabilized with a bolus of hypertonic saline (Braun Vetcare hypertonic NaCl solution 7.5%®) and interruption of PIRRT. Following the return of extracorporeal blood to the patient and hypertonic saline administration, this patient regained consciousness and became normotensive. Median blood pressure in all dogs during PEX was 140 mmHg (range 60–220). Two dogs (case 4 and 6) required additional blood transfusions with pRBC. The PEX therapy was performed the day following admission at our center in 4/6 dogs (case 2, 4, 5, and 6) and on day 2 of hospitalization in 2/6 dogs.

### Outcome

Median length of hospitalization until either discharge from hospital or time of death was 5.5 days (range 4–23 days). Four dogs (case 3, 4, 5, 6) were euthanized at the owners' request due to lack of clinical improvement, ongoing costs and grave prognosis. A post-mortem examination was performed in all non-surviving dogs and the results were characteristic of CRGV as described by Holm et al. (5), with renal glomerular and tubular necrosis and vessel occlusion with thrombi.

Of the 4 non-survivors (case 3, 4, 5, and 6), median time to euthanasia was 5 days (4–6 days). Case 3 continued with severe clinical deterioration despite repeat cycles with both PEX and PIRRT. Her clinical deterioration consisted of obtundation, worsening azotemia, marked pelvic limb vasculitis with the concern that she would not tolerate the required PIRRT cycles for management of her AKI. Therefore, the owner elected euthanasia. Case 4 was euthanized due to neurological deterioration with ongoing seizure activity; post-mortem examination showed multifocal neuronal degeneration and necrosis with hemorrhage and edema. Case 5 developed septic shock and hypoxemia; post-mortem examination revealed the presence of severe pneumonia and bacterial endocarditis. This patient had a sudden clinical deterioration following the second PEX cycle, which was performed 48 h after placement of the dialysis catheter. Echocardiography in this patient was performed prior to catheter placement and did not reveal any signs of endocarditis or systolic dysfunction. Case 6 was euthanized due to sudden development of respiratory failure requiring further life-support with mechanical ventilation; heart failure and volume overload were excluded based on physical examination, patient-side echocardiography and thoracic radiographs. The owners elected euthanasia and a cause for the respiratory distress was not identified despite post-mortem examination. There were no significant pathological changes on histological examination of the heart and lungs of this patient.

Two dogs (case 1 and 2) survived to discharge from hospital. The survivors were hospitalized for 10 and 23 days, respectively. During hospitalization both dogs were hypertensive and anuric. Case 1 received 1 PEX cycle, and case 2 received 2 PEX cycles without major complications. One of the survivors (case 2) became polyuric after starting diuretic therapy. The patient remained polyuric throughout its single PEX cycle and was weaned off diuretics 19 h post PEX. At the time of discharge, case 1 had mild azotemia (creatinine 202 μmol/L, urea 18.3 mmol/L) and hyperphosphatemia (2.11 mmol/L, RI 0.8–1.6 mmol/L). This was an improvement compared with pre-treatment values [creatinine 760 μmol/L (RI 59–138 μmol/L), urea 55.1 mmol/L (RI 3–9.1 mmol/L) and phosphorus 3.68 mmol/L (RI 0.8–1.6 mmol/L)]. The second survivor (case 2) also had mild azotemia at time of discharge (creatinine 257 μmol/L, urea 17.2 mmol/L, and a normal phosphorus 1.92 mmol/L) compared to a moderate azotemia while hospitalized (creatinine 351 μmol/L, urea 30.1 mmol/L and phosphorus 3.64 mmol/L). These dogs were still alive at the time of last follow up 3 years and 2 months (case 1) and 1 year and 9 months (case 2), respectively after discharge from hospital. By that time, the dogs were doing well clinically with no requirements for further medical management. The azotemia had completely resolved for case 1, whilst case 2 had a slightly increased urea (9.2 mmol/L) but a normal creatinine concentration (101 μmol/L). Both cases were normotensive and were considered as IRIS Chronic Kidney Disease stage 1 (21). During hospitalization, they both had and IRIS grade III AKI.

## Discussion

This study describes the use of PEX in 6 dogs clinically diagnosed with CRGV. Unfortunately, only 2/6 dogs survived to discharge. Overall, PEX was tolerated by dogs; however, commonly reported complications of apheresis therapy were identified. Current human recommendations for the treatment of TMA would be to perform daily cycles for three days, though this approach does not have strong evidence base (11). The decision to perform a further treatment with a cycle of PEX or PIRRT 24–48 h later was made following consideration of patient stability, clinical progression and patient- and owner-related factors, including financial considerations. Ultimately, the role that PEX played in either the reduced progression or recovery in the two surviving dogs cannot be ascertained from this study. The two survivors appeared to respond to cycles of PEX therapy as they became less clinically affected with no development of neurological abnormalities compared to the non-survivors. Given the potential of similarities in etiology for TMA between dogs and people and the high mortality rate reported in dogs with CRGV, PEX could be considered a viable modality of therapy in these dogs, although further research is warranted.

Thrombotic microangiopathy in people can occur secondary to several causes, including TTP and HUS. A detailed review of the causes of TMA in people is beyond the scope of this study and readers are referred elsewhere (1, 6, 7). In brief, TTP is a hypercoagulable state, caused by inhibition of ADAMTS-13, a factor-cleaving protease, by auto-antibodies which leads to a lack of degradation of von Willebrand factor multimers. Von Willebrand factor multimers are prothrombotic, thus ADAMTS13 inhibition leads to microthrombi formation, microangiopathic hemolytic anemia, thrombocytopenia and multiple organ injury, including renal injury (24). In people with TTP, PEX has demonstrated to substantially increase survival from 10 to 78% and is now considered a first-line treatment, even in cases with uncertain diagnosis (1, 8).

Hemolytic Uremic Syndrome, on the other hand, can be caused by detectable underlying factors, such as Shiga toxin-producing *E. Coli* or drugs (e.g., quinine or calcineurin inhibitors) (1). Management in these cases consists of addressing the trigger for the condition when known. The term aHUS describes a condition typically seen in pediatric patients, characterized by TMA (microangiopathic hemolytic anemia, thrombocytopenia and renal failure) but without the prodromic hemorrhagic diarrhea (25). Atypical HUS seems to be caused by complement-mediated microangiopathy, resulting from an uncontrolled activation of the alternative complement pathway (1, 26); the main clinical feature is kidney failure (1, 8). Anti-complement therapy has been recently shown to be the most effective therapy in these patients. However, the cost of the treatment is prohibitive for veterinary patients (25, 27). For both these conditions, PEX is considered a standard treatment and has been shown to reduce mortality (1, 8, 26, 28).

Therefore, the major goals of PEX in TTP are the removal of the antibodies against ADAMTS-13 and the supplementation of ADAMTS-13 via FFP, preventing further thrombi formation. Similarly, in aHUS PEX can remove the aberrant complement and provide replacement with normal complement factors (28). In people with TMA, PEX with FFP has shown a reduction in mortality compared to single plasma infusion alone (7, 29). Because of the high mortality rate associated with TTP, PEX is often initiated in the presence of an unexplained thrombocytopenia and anemia secondary to suspected microangiopathic disease even before confirmation of this condition (7).

In dogs, reported causes for TMA include HUS (30) and CRGV (2–5), with the latter being first reported in the USA in 1988 and initially referred to as “Alabama Rot” (2, 3). In the outbreaks reported in the USA, most of the dogs affected were greyhounds who developed a variable degree of the disease, including cutaneous lesions, localized specifically to the limbs, and AKI. Not all dogs developed AKI and some patients developed AKI before the cutaneous lesions could be identified, making the clinical presentation heterogeneous (2). It is unclear whether the previously reported “Alabama Rot” represents the same disease as the CRGV that is affecting dogs in the United Kingdom or whether a different etiopathogenesis exists. Possible differential diagnoses for the underlying cause of AKI include Leptospirosis which can be tested for by serology, PCR and histopathology of renal and hepatic tissue (5). Not all clinical presenting features in patients with leptospirosis are comparable with the presentation of CRGV cases (e.g., skin lesions are not normally reported with leptospirosis). Another differential could include an adverse drug reaction causing skin lesions and multiple organ dysfunction syndrome. However, CRGV cases develop skin lesions first and typically have not had any exposure to drugs until after these lesions appear. Another known cause leading to TMA in dogs other than CRGV is HUS, though skin lesions have previously never been reported in canine HUS. In human HUS, the most commonly reported forms are caused by *E. coli* or *Shigella dysenteriae* Shiga toxin or by *Salmonella* or *Campylobacter* infections, which can be identified by fecal culture (5). To the authors' knowledge, there appears to be no other obvious differential diagnosis for the combination of clinical signs in the cases presented in this study. No underlying causative agent has been identified in dogs with CRGV despite a detailed investigation that included the search for immune-complex deposition, viral (canine circovirus) or bacterial disease presence (Shiga toxin, *E.coli, Leptospira*) (2, 3, 5) albeit performed in relatively small numbers of cases and with the caveat that chronology may be of importance in terms of sampling and identifying these factors. Other causes, such as ADAMTS-13 deficiency, complement-activation or detection of toxins responsible for TMA in people have not been investigated thus far.

All patients described in this study developed cutaneous lesions, with evidence of microangiopathic damage, thrombocytopenia and AKI with 4/6 dogs also developing anemia. Renal biopsy was available for confirmation of CRGV in 4/6 dogs. In the remaining 2 dogs clinicopathological features, combined with compatible dermal histopathology, strongly indicated that CRGV was the underlying disease process despite the lack of renal biopsies. Definitive ante-mortem confirmation of TMA in the 2 surviving dogs would have required renal biopsy, but the risk of potential bleeding in the face of thrombocytopenia was considered to outweigh the benefit of confirming the diagnosis.

At the time of manuscript preparation, no treatment has been demonstrated to reduce the high mortality associated with this disease in dogs developing oliguric or anuric AKI (5). The cases described in this report presented with anuric or oliguric AKI and rapidly deteriorating conditions that failed to respond to medical treatment. Thus, due to the similarity of clinical progression and clinic-pathological abnormalities resembling either TTP or aHUS in people, we postulated that PEX would be a reasonable method of treating these cases.

The decision to perform PEX in the dogs included in this study was made when there was an apparent progression of their CRGV syndrome, with the aim of intervening early to achieve removal of suspected immune complexes that could be involved in the pathophysiological mechanism of CRGV. Nevertheless, the patients included in this study were already at an advanced stage in the disease process on the basis that they presented with azotemia and oligoanuria. The optimal time to intervene with therapies such as PEX has not been established. However, from clinical experience, intervention after the development of severe AKI appears to be associated with a guarded prognosis. This raises questions as to whether the most appropriate time to intervene with therapies such as PEX is at the time point when non-azotemic AKI is identified or UOP first begins to fall. However, lack of a definitive diagnostic test, other than renal biopsy, the intense and invasive nature of PEX therapy that is not without the potential for complications, makes this a challenging judgment call to make, particularly considering that based on clinical experience some of the less severely affected but clinically suspected cases may show improvement with standard management for AKI. In light of the poor prognosis associated with this condition, once AKI and oliguria develop, we considered PEX to be the best alternative to manage this disease as long-term dialytic therapy was not a clinically or financially viable option for these dogs currently in the UK. These cases were particularly severely affected. It is possible that patients with CRGV can present with a spectrum of clinical signs, some of which may be azotemic and managed in primary care practice recovering with supportive treatment and not reaching the level of severity of the disease of those cases that make it to referral practice.

The role that PEX played in the positive outcome of 2 of the 6 cases is difficult to ascertain and represents a major limitation of this report. It is possible that these patients would have improved in terms of their clinical status with continued supportive therapy, particularly given that there was indication of improved UOP after furosemide administration in one of the surviving cases. As previously reported, a positive response in UOP following furosemide administration in patients with AKI may reflect less severe renal injury (31). Nevertheless, the potential utility of this modality of therapy for this indication requires further investigation. In light of the fact that patients with CRGV and oliguric AKI have such a poor prognosis, the positive outcome in the two cases presented is of interest.

All of the 4 non-survivors (case 3, 4, 5, and 6) were euthanized at their owner's request within the first week of hospitalization and often requested the day following a PEX cycle. There were various reasons for the decision for euthanasia including clinical deterioration, grave prognosis and costs. All owners were aware of the timeframes expected for renal recovery, the need of repeat cycles as well as the financial investment these advanced therapeutic options require from the outset. Given the complexity of these cases, various factors may have influenced some of the clients to request humane euthanasia instead of continuing with management early in the course of the disease.

Extracorporeal therapy requires specially trained staff, clinical expertise and special equipment. PEX is not a risk-free therapy, but studies in people have shown that there can be improved survival rates with the use of PEX in TMA patients (10) and that adverse effects associated with PEX have been reported to be <10% (27). Some form of quantitative evaluation of the efficacy of PEX therapy would have been useful to assess response to therapy in individual patients. Future studies, should focus on whether systemic markers of inflammation could be used for these purposes, for example C-reactive protein.

The complications associated with PEX observed in the current study are similar to those previously reported, such as hypotension and citrate-induced hypocalcemia (12–18). Other complications of extracorporeal therapies previously reported such as hypersensitivity reaction to replacement fluids (16) were not observed in our study. One of the non-survivors (case 5) developed septic shock and hypoxemia following her second PEX cycle; post-mortem examination revealed the presence of bacterial endocarditis and pneumonia. It is unclear whether a catheter-related infection or aspiration were the cause of the rapid deterioration. The owners elected euthanasia following the development of this deterioration. The other 3 non-survivors (case 3, 4, and 6) did not have any documented complications after the PEX therapy. Case 3 and 6 both developed severe hypotension during a PIRRT cycle (URR 45.6%) and PEX cycle (1.8 PV exchanged), respectively. They were the two smallest dogs in this population (10.4 and 15 kg, respectively) and it is hypothesized that hypovolemia contributed to the reported hypotension. Both the rate of extracorporeal therapy and size of extracorporeal circuit used may have contributed to hypovolemia. In particular, case 6 was successfully weaned off vasopressors after returning blood from the extracorporeal circuit and transfusion of a unit of pRBC suggesting that hypovolemia may well have been a significant contribution to the hypotension. The neurological signs case 6 experienced were likely caused by reasons other than an ischemic insult due to rapid improvement following stabilization. Case 3 also clinically improved at time of ending cycle and returning blood to patient with improvement of perfusions parameters.

Previous studies have reported that glomerular disease often leads to a UPC ratio >2.0 in dogs (32). Renal loss of albumin can lead to hypoalbuminemia which was seen in 5/6 dogs in our study. Moreover, as albumin is a negative acute phase protein, the inflammatory nature of this disease could have contributed further to decreased albumin concentration. As albumin is the main determining factor of colloid oncotic pressure, marked decreases in albumin concentration could negatively impact fluid distribution between body compartments (32). The dogs in the present study had marked proteinuria, median UPC ratio of 2.95 (range 0.49–11.16) with hypoalbuminemia reported in 5/6 dogs. The albumin concentrations of the two dogs that demonstrated hypotension were 18.6 g/L for case 3 and 30.4 g/L for case 6, respectively. These values were not deemed severe enough to cause reduced oncotic pressure and therefore it is unlikely that in either of these patients hypotension was the result of third spacing due to hypoalbuminemia. Both dogs responded well to medical management with vasopressor therapy (noradrenaline). Case 6 also developed clinical signs of hypocalcemia (facial twitching) with an ionized calcium of 0.67 mmol/L (RI 1.13–1.33 mmol/L) which responded to calcium gluconate supplementation. The hypotension was only experienced in PEX cycle one; for the second cycle we decreased the calculated PV from previous 1.8–1.4 which was tolerated well. This patient was later euthanized at the owner's request due to clinical deterioration and onset of respiratory distress. Case 3 which developed hypotension during PIRRT was later euthanized at the owner's request due to a combination of welfare issues, requirement for repeat cycles of PIRRT with clinical deterioration, ongoing financial commitment and grave prognosis for survival.

Two dogs (case 3 and 6) developed tachycardia during PEX therapy. Case 6, as described above, became hypotensive possibly secondary to hypovolemia which may explain the compensatory tachycardia. However, case 3, was normotensive throughout the PEX cycle and as such it seems unlikely that volume changes contributed to tachycardia. Case 3 did, however, become agitated and required additional sedation with opioid analgesia and benzodiazepine constant rate infusion which improved the tachycardia. As PEX therapy is an invasive procedure, this requires patients to be calm during the cycle to allow good blood flow and avoid catheter occlusion related issues.

In total, three dogs (case 3, 4, and 6) developed neurological abnormalities. Case number 3 and 6 developed neurological signs with abnormal mentation during PIRRT and PEX therapy respectively due to hypovolemia. Case 4 already had abnormal neurological signs prior to PEX with seizure activity at the time of presentation. It is difficult to ascertain if these were a consequence of the clinical progression of the disease; neurological post-mortem examination was performed in case 4 and documented evidence of CNS necrosis with hemorrhages.

The current recommendation in human medicine regarding PEX therapy is to perform 1–1.5 PV exchange during a cycle (11). The extracorporeal unit (Prismaflex) calculates the exchanged plasma volume and does not include the pre-blood pump (citrate) flow rate in this volume. This led to a wide range of PV exchanged (range 1–3.3) in the retrospective analysis. In each case, initial PV calculations were performed for a 1–1.5 PV exchange. Our median PV exchanged was 1.8 which could have contributed to the complications experienced by our patients. As discussed earlier our wide range of PV exchanged includes the pre-blood pump. However, there are few conditions in people where a large extracorporeal volume exchange is recommended (e.g., immune-mediated thrombocytopenia, overdose, envenomation and poisoning) (11).

The non-survivors that did not benefit from PEX may have presented at later stages of the disease where renal function was irreversibly compromised. In such patients, repeated cycles of PEX combined with hemodialysis or PIRRT may have been necessary in order to achieve a successful outcome. In people with TMA, and specifically TTP, repeated cycles are often necessary and recovery times often extend to weeks (1, 8, 33).

However, 3 out of 4 non-survivors (case 4, 5, 6) were ultimately euthanized due to co-morbidities that occurred in addition to their renal injury; namely neurological deterioration, pneumonia/endocarditis and respiratory failure. It can be argued that the number of cycles of PEX performed in the non-surviving dogs was insufficient to allow appropriate renal recovery and/or that these patients should have been supported with renal replacement therapies e.g., hemodialysis in order to reduce the morbidity. However, this was not possible in the patients described in this study. In addition, in the cases reported, the decision to perform euthanasia was not limited to renal recovery.

This study has some inherent limitations, such as the small number of dogs included and the lack of a control group. Holm et al. (5) retrospectively reviewed a population of dogs with clinical signs consistent with CRGV and a confirmation of TMA by renal histopathology. None of those dogs (*n* = 30) received PEX as a therapeutic option but three dogs received PIRRT. Sadly, all 30 dogs died or were euthanized. However, Holm et al. (5) also report that they had clinical data in 6 surviving dogs with suspected CRGV, which responded well to symptomatic management but that were excluded from their study due to lack of confirmation of CRGV by renal histopathology. The fact that dogs with CRGV present at differing stages of their disease that vary in severity and that the diagnosis can only be confirmed by performing an invasive diagnostic test i.e., renal histopathology, severely limits our ability to perform comparative clinical studies in dogs with this disease. The development of a CRGV illness severity score could help mitigate some of these challenges. For patients that are not thrombocytopenic but where there is a high index of suspicion for CRGV, renal biopsy may be considered in order to confirm the diagnosis. It is difficult to ethically justify this however, given that there are no known successful treatment options other than supportive treatment for patients with confirmed CRGV.

Future prospective randomized studies comparing dogs with CRGV treated with and without PEX and stratified by illness severity score would be needed to assess therapeutic efficacy. Additional studies evaluating for the presence of autoantibodies to ADAMTS13 in dogs with CRGV, as observed in people with TTP, would also be recommended to clarify the pathogenesis of this disease.

Another limitation regarding PEX therapy in this study included the available fluids for replacement and the lack of guidelines indicating the optimal timing of plasma infusion initiation. Alternative fluids, such as synthetic colloids might have been beneficial for these cases although concerns regarding their safety is still unknown, especially in patients with AKI (34). Nevertheless, we can only speculate that outcome may have been different whether given more time or greater number of treatment options either with PEX or PIRRT. The results of renal histopathology in the four non-surviving dogs indicated a severe and disseminated disease, suggesting that chances of renal recovery were likely to be limited.

## Conclusions

In this retrospective study, we have described the novel application of PEX as an adjunctive treatment in dogs with severe CRGV. Plasma exchange is an invasive procedure, requiring special equipment and specially-trained staff. However, the good outcome in the two survivors, in light of the high mortality reported with CRGV, warrants further consideration whether PEX could prove helpful in selected cases. Moreover, our finding should encourage further studies aimed at better understanding the pathogenesis of this condition, and further evaluation of the potential role of PEX in the treatment of affected dogs.

## Ethic statement

This study was approved by the Clinical Research Ethical Review Board. URN: M2016 0086.

## Author contributions

All authors listed have made a substantial, direct and intellectual contribution to the work, and approved it for publication.

### Conflict of interest statement

The authors declare that the research was conducted in the absence of any commercial or financial relationships that could be construed as a potential conflict of interest. The reviewer AS and handling Editor declared their shared affiliation.

## Abbreviations

AKI: Acute Kidney Injury
aHUS: atypical HUS
CRGV: Cutaneous and Renal Glomerular Vasculopathy
HUS: Hemolytic Uremic Syndrome
MAT: Macroscopic Agglutination Test
pRBC: Packed Red Blood Cells
PEX: Plasma Exchange
PIRRT: prolonged intermittent renal replacement treatment
PV: plasma volume
RI: reference interval
TMA: Thrombotic Microangiopathy
TPE: Total Plasmapheresis
TTP: Thrombotic Thrombocytopenic Purpura
UOP: urine output
UPC: urine protein creatinine ratio.

## References

[1] George JN Nester CM. Syndromes of thrombotic microangiopathy. N Engl J Med. (2014) 371:654–66. 10.1056/NEJMc1410951 25119611

[2] Carpenter JL Andelman NC Moore FM King NW Jr. Idiopathic cutaneous and renal glomerular vasculopathy of Greyhounds. Vet Pathol. (1988) 25:401–7. 10.1177/030098588802500601 3212884

[3] Hertzke DM Cowan LA Schoning P Fenwick BW. Glomerular ultrastructural lesions of idiopathic cutaneous and renal glomerular vasculopathy of greyhounds. Vet Pathol. (1995) 32:451–9. 10.1177/030098589503200501 8578634

[4] Rotermund A Peters M Hewicker-Trautwein M Nolte I. Cutaneous and renal glomerular vasculopathy in a great dane resembling “Alabama Rot” of greyhounds. Vet Rec. (2002) 151:510–2. 10.1136/vr.151.17.510 12431001

[5] Holm LP Hawkins I Robin C Newton RJ Jepson RE Stanzani G. Cutaneous and renal glomerular vasculopathy (CRGV) causing acute kidney injury (AKI) in dogs in the United Kingdom (UK). Vet Rec. (2015) 176:384–95. 10.1136/vr.102892 25802439 PMC4413843

[6] Ruggenenti P Noris M Remuzzi G. Thrombotic microangiopathy, hemolytic uremic syndrome, and thrombotic thrombocytopenia purpura. Kidney Int. (2001) 60:831–46. 10.1046/j.1523-1755.2001.060003831.x 11532079

[7] Clark WF. Thrombotic microangiopathy: current knowledge and outcomes with plasma exchange. Semin Dial. (2012) 25:214–9. 10.1111/j.1525-139X.2011.01035.x 22309967

[8] Stevenson ME Leung N Winters JL. What are the newer applications for therapeutic apheresis in nephrology? Semin Dial. (2016) 29:350–3. 10.1111/sdi.12522 27472247

[9] Williams ME Balogun RA. Principles of separation: indications and therapeutic targets of plasma exchange. Clin J Am Soc Nephrol. (2014) 9:181–90. 10.2215/CJN.04680513 24178973 PMC3878701

[10] Peigne V Perez P Resche Rigon MR Marioette E Canet E Mira JP. Causes and risk factors of death in patients with thrombotic microangiopathies. Intens Care Med. (2012) 38:1810–7. 10.1007/s00134-012-2638-5 22797353

[11] Schwartz J Padmanabhan A Aqui N Balogun RA Connolley-Smith L Denelaney M. Guidelines on the use of therapeutic apheresis in clinical practice – evidence-based approach from the writing committee of the american society for apheresis: the seventh special issue. J Clin Apher. (2016) 31:149–338. 10.1002/jca.21470 27322218

[12] Matus RE Scott RC Saal S Gordon BR Hurtvitz AI. Plasmapheresis-immunoadsorption for treatment of systemic lupus erythematosus in a dog. J Am Vet Med Assoc. (1983) 182:499–502. 6339455

[13] Matus ER Leifer CE Gordon BR eMacEwen EG Hurtvitz AI. Plasmapheresis and chemotherapy of hyperviscosity syndrome associated with monoclonal gammopathy in the dog. J Am Vet Med Assoc. (1983) 183:215–8. 6885594

[14] Lippi I Perondi F Ross SJ Marchetti V Lubas G Guidi G. Double filtration plasmapheresis in a dog with multiple myeloma and hyperviscosity syndrome. Open Vet J. (2015) 5:108–12. 26623375 PMC4663801

[15] Bartges JW Klausner JS Bostwick EF Hakala JE Lennon VA. Clinical remission following plasmapheresis and corticosteroid treatment in a dog with acquired myasthenia gravis. J Am Vet Med Assoc. (1990) 196:1276–8. 2332375

[16] Crump KL Seshandri R. Use of the therapeutic plasmapheresis in a case of canine immune-mediated hemolytic anemia. J Vet Emerg Crit Care (2009) 19:375–80. 10.1111/j.1476-4431.2009.00431.x 25164638

[17] Walton S Ryan KA Davis JL Acierno M. Treatment of meloxicam overdose in a dog via therapeutic plasma exchange. J Vet Emerg Crit Care (2017) 27:444–50. 10.1111/vec.12607 28481472

[18] Walton S Ryan KA Davis JL Acierno M. Treatment of ibuprofen intoxication in a dog via therapeutic plasma exchange. J Vet Emerg Crit Care (2017) 27:452–7. 10.1111/vec.12608 28481451

[19] Tovar T Deitschel S Guenther C. The use of therapeutic plasma exchange to reduce serum bilirubin in a dog with kernicterus. J Vet Emerg and Crit Care (2017) 27:458–64. 10.1111/vec.12622 28605161

[20] Brown S Atkins C Bagley R Carr A Cowgill L Davidson M. Guidelines for the identification, evaluation, and management of systemic hypertension in dogs and cats. J Vet Intern Med. (2007) 21:542–58. 10.1111/j.1939-1676.2007.tb03005.x 17552466

[21] International Renal Interest Society (IRIS) (2016) Available online at: http://www.iris-kidney.com/guidelines/index.html (Accessed March 28, 2018).

[22] Schuler S Francey T Hartmann K Hugonnard M Kohn B Nally JE. European consensus statement on leptospirosis in dogs and cats. J Small Anim Pract. (2015) 56:159–79. 10.1111/jsap.12328 25754092

[23] Langston C Poeppel K Mitelberg E Eatroff A editors. Veterinary dialysis handbook, extracorporeal renal replacement therapies: intermittent hemodialysis and continuous renal replacement therapy. 6th Advanced Renal Therapies Symposium; 2014 Mar 26-29; Animal Medical Center, New York, NY: Nephrology Knowledge: 2014 Mar. 51-52p.

[24] Rock GA Shumak KH Buskard NA Blanchette VS Kelton JG Nair RC. Comparison of plasma exchange with plasma infusion in the treatment of thrombotic thrombocytopaenic purpura. N Engl J Med. (1991) 325:393–7. 10.1056/NEJM199108083250604 2062330

[25] Tsai HA Mechanistic approach to the diagnosis and management of atypical haemolytic uremic syndrome. Transfus Med Rev. (2014) 28:187–97. 10.1016/j.tmrv.2014.08.004 25280590

[26] Neyrink MM Vrielink H. Calculations in apheresis. J Clin Apher. (2015) 30:38–42. 10.1002/jca.21347 25041907

[27] Legendre GG Nichols WC Muus P Greenbaum LA Babu S Bedrosian C Terminal complement inhibitor eculizumab in atypical haemolytic-uremic syndrome. N Engl J Med. (2013) 325:2169–81. 10.1056/NEJMoa1208981 23738544

[28] Hans R Sharma RR Marwaha N Suri D Kumar R Gupta A. Efficacy and safety of therapeutic plasma exchange by using apheresis devices in pediatric atypical hemolytic uremic syndrome patients. J Clin Apher. (2016) 31:381–7. 10.1002/jca.21412 26212115

[29] Micheal M Elliot EJ Ridley GF Hodson EM Craig JC Interventions in hemolytic uremic syndrome and thrombotic thrombocytopenic purpura. Cochrane Database Syst Rev. (2009) CD003595. 10.1002/14651858.CD003595.pub2 PMC7154575 19160220

[30] Holloway S Senior D Roth L Tisher CC. Hemolytic uremic syndrome in dogs. J Vet Intern Med. (1993) 7:220–7. 10.1111/j.1939-1676.1993.tb01011.x 8246211

[31] Ho KM Power BM. Benefit and risks of furosemide in acute kidney injury. Anaesthesia (2010) 65:283–93. 10.1111/j.1365-2044.2009.06228.x 20085566

[32] Harley L Langston C. Proteinuria in dogs and cats. Can Vet J. (2012) 53:631–8. 23204582 PMC3354822

[33] Zou X Wu T Zhang X Qu A Tian S. Thrombotic thrombocytopenic purpura (TPP) successfully, rescued by plasma exchange in the ICU: a report of two cases. Exp Ther Med. (2016) 12:329–32. 10.3892/etm.2016.3265 27347058 PMC4907122

[34] Hayes G Benedicenti L Mathews K. Retrospective cohort study on the incidence of acute kidney injury and death following hydroxyethyl starch (HES 10% 250/0.5/5:1) administration in dogs (2007-2010). J Vet Emerg Crit Care (2016) 26:35–40. 10.1111/vec.12412 26587795

